# Low bone mass in microscopic colitis

**DOI:** 10.1186/1471-230X-11-58

**Published:** 2011-05-19

**Authors:** Katalin Lőrinczy, Gábor Lakatos, Katalin Müllner, István Hritz, Péter László Lakatos, Zsolt Tulassay, Pál Miheller

**Affiliations:** 1 2nd Department of Medicine, Semmelweis University, Budapest, Hungary

**Keywords:** bone, microscopic colitis, Crohn's disease, osteoporosis

## Abstract

**Background:**

Microscopic colitis presents with similar symptoms to classic inflammatory bowel diseases. Osteoporosis is a common complication of Crohn's disease but there are no data concerning bone metabolism in microscopic colitis.

**Aims:**

The aim of the present study was to evaluate bone density and metabolism in patients with microscopic colitis.

**Methods:**

Fourteen patients microscopic colitis were included in the study, and 28 healthy persons and 28 age and gender matched Crohn's disease patients were enrolled as controls. Bone mineral density was measured using dual x-ray absorptiometry at the lumbar spine, femoral neck and the radius. Serum bone formation and bone resorption markers (osteocalcin and beta-crosslaps, respectively) were measured using immunoassays.

**Results:**

Low bone mass was measured in 57.14% patients with microscopic colitis. Bone mineral density at the femoral neck in patients suffering from microscopic colitis and Crohn's disease was lower than in healthy controls (0.852 ± 0.165 and 0.807 ± 0.136 vs. 1.056 ± 0.126 g/cm^2^; p < 0.01). Bone mineral density at the non-dominant radius was decreased in microscopic colitis patients (0.565 ± 0.093 vs. 0.667 ± 0.072 g/cm^2^; p < 0.05) but unaffected in Crohn's disease patients (0.672 ± 0.056 g/cm^2^). Mean beta-crosslaps concentration was higher in microscopic colitis and Crohn's disease patients than controls (417.714 ± 250.37 and 466.071 ± 249.96 vs. 264.75 ± 138.65 pg/ml; p < 0.05). A negative correlation between beta-crosslaps concentration and the femoral and radius t-scores was evident in microscopic colitis patients.

**Conclusions:**

Low bone mass is frequent in microscopic colitis, and alterations to bone metabolism are similar to those present in Crohn's disease. Therefore, microscopic colitis-associated osteopenia could be a significant problem in such patients.

## Background

Microscopic colitis (MC) is defined by chronic, watery diarrhoea, abdominal pain and weight loss. However, macroscopically normal colonic mucosa is evident on radiological and endoscopic examination, and microscopic examination is required for the detection of diagnostic histopathological features [1,2]. MC normally occurs in middle-aged patients, with a peak incidence in individuals aged approximately 65 years. The annual incidence of MC is between four and six per 100,000 individuals. However, the significance of these morphologically distinct diseases is underestimated in daily clinical practice and the pathogenesis underlying MC has yet to be elucidated. Abnormal collagen metabolism [3], bacterial toxins [4] and drugs [5] could be responsible for mucosal injury in MC, although the colonic mucosa appears normal on colonoscopy examination and diagnosis is established using histology.

Two types of MC were initially described more than 30 years ago [6,7]. Collagenous colitis (CC) is defined by a sub-epithelial collagen layer wider than 10 μm [8]. Diagnostic criteria for lymphocytic colitis (LC) are more than 10 [9] or 20 [10] intraepithelial lymphocytes (IEL)/100 epithelial cells of the colonic mucosa. Microscopic colitis is thought to be a multifactorial disease but the exact cause is unknown. A high incidence in families suggests a degree of genetic susceptibility [11,12]. There is a high incidence of spontaneous resolution of symptoms, and budesonide is the drug of choice for first-line treatment in patients with severe symptoms [13].

A relationship between MC and members of the classic inflammatory bowel disease (IBD) group including Crohn's disease (CD) and ulcerative colitis (UC) is based on epidemiological, pathological and clinical associations. Several case reports demonstrate that MC can progress to IBD [14-16]. However, a large retrospective analysis demonstrated no association between the presence of MC and progression to IBD [9]. Olesen et al. demonstrated that 12% of patients with LC reported a family history of other bowel disorders including IBD, celiac disease and CC [17].

Decreased bone mineral density (BMD) is a common complication of IBD and is present in 30-77% of cases [18,19], predominantly in patients suffering from CD [20]. Compared to healthy controls, individuals suffering from CD have a relative risk of vertebral and hip fracture of 1.59 and 1.72, respectively [21]. Recent data indicate that low bone mass associated with CD correlates with the basic pathology of CD rather than malabsorption or complications of steroid treatment [22,23].

There are currently no studies concerned with possible alterations in bone metabolism in MC patients. The aim of this study was to evaluate the bone density and bone metabolism in patients with MC. The secondary aim was to compare the alterations in bone metabolism of MC patients with bone metabolism in CD patients and healthy controls.

## Methods

### Patient selection

Fourteen MC patients (12 women and two men with a mean age of 49.79 ± 13.06 years) were included in the study. Ten were diagnosed with LC and four with CC. Enrolment criteria included asymptomatic patients who had not taken medication for six weeks prior to commencement of the study. Those patients who had been subjected to treatment with budesonide for longer than eight weeks, or within six weeks prior to enrolment in the study, were excluded from participating. The short plasma half-life of budesonide (1.5-3.5 hours) [24], the 24 hours duration of effects and the first pass effects of the drug were taken into consideration to define the wash-out period. Remission during MC was defined as two or less bowel movements per day without taking medication. Remission had been achieved using budesonide during the history of these patients; none of the enrolled patients had been previously treated with systemic steroids. The presence of celiac disease was excluded using serology (tissue transglutaminase and endomysial antibody) and duodenal histology. Lymphocytic colitis was defined as more than 10 IELs/100 epithelial cells situated in the mucosa, and the diagnostic criterion for CC was a subepithelial collagen layer wider than 10 μm.

### Control groups

Twenty-eight healthy persons (HC) and 28 CD patients matched for age, gender and postmenopausal state were enrolled as controls. CD patients were in remission as defined by the Crohn's disease activity index (CDAI < 150) [25], and had been steroid free for one year. Azathioprine up to 2.5 mg/body weight kg and mesalazine up to 4 g daily was permitted as a maintenance therapy for CD patients. None of the participants had the stricturing or penetrating (Vienna Classification B2 or B3) types of the disease [26]. The members of the HC group were matched for age, gender and menopausal status to the MC patients. Subjects provided written informed consent, and the study protocol was approved by the regional and institutional committee of science and research ethics (SE-106/2007).

Demographical data, significant medical history (localization and duration of disease, surgical history and drug use), risk factors for osteoporosis (body mass index, menopausal state, smoking status, family history for osteoporosis and previous low trauma bone fracture) were recorded. Patients taking medications that affected vitamin K metabolism were not eligible to participate in the study.

### Bone mineral density measurements

Bone mineral density (BMD) measurements were performed using dual-energy X-ray absorptiometry of the lumbar spine (L2-L4), the left femoral neck and the non-dominant radius using a Hologic QDR 4500C instrument (Hologic, Waltham, MA). For analysis, v. 9.03D software was used. Z-scores were calculated according to the manufacturer's reference curves (the number of standard deviations (SD) from age- and sex-matched healthy controls). The third National Health and Nutrition Examination Surveys (NHANES III) normative data were used as a reference database for femoral bone density measurements. World Health Organization (WHO) criteria for low BMD were applied for this analysis [27]. Low bone mass and osteoporosis were defined as a BMD *t*-score below -1 and below -2.5, at the lumbar spine or the femoral neck, respectively. Quality control was maintained by carrying out daily scanning of an anthropometric spine phantom. The coefficient for the variation of BMD measurements on the spine phantom over a period of four years was 0.35%.

### Parameters of bone metabolism

Serum calcium (normal: 2.25-2.61 mmol/l), parathyroid hormone (normal: 10-65 pg/ml) and thyroid stimulating hormone (normal: 0.3-3.3 mU/L) levels were determined before the study commenced to exclude the presence of other types of metabolic bone diseases.

Fasting blood samples for evaluating markers of bone metabolism were taken between 7 and 8 a.m. Osteocalcin (OC) is a bone-specific calcium binding protein produced by osteoblasts during bone synthesis, and serves as a good marker for bone formation. After release, it accumulates in the bone matrix and proportional amounts leak into the blood stream [28]. More than 90% of the organic bone matrix consists of type I collagen. During certain physiological and pathological processes, a degradation product of mature type I collagen, beta-crosslaps (bCL), is released into the bloodstream. Accordingly, bCL is a useful marker for monitoring the process of bone resorption [29].

Serum OC and bCL levels were measured using an electrochemiluminescence immunoassay (Elecsys N-MID Osteocalcin and Elecsys b-CrossLaps, Roche, Nutley, NJ). The OC immunoassay detects a stable 43 amino acid fragment of the N-terminal end (N-MID fragment). The immunoassay was based on monoclonal antibodies against epitopes located on the stable fragment. The normal concentrations in the serum were 0-320 pg/ml for bCL and 20-48 ng/ml for OC.

### Statistics

Calculations were performed using SPSS statistics 15.0 software. Paired and independent sample Student's *t*-tests, Pearson correlations and chi-square tests were applied. The results were presented as mean ± SD. Results were considered significant when p < 0.05.

## Results

Major clinical characteristics of the age, gender and postmenopausal status matched groups are presented in Table 1.

**Table 1:** Clinical characteristics of patient groups.

	Microscopic colitis (10 LC and 2 CC)		Crohn's disease
duration of disease (years)	4.33 ± 1.66	ns.	5.03 ± 3.53

actual symptoms (number of liquid stools/day)	1.41 ± 0.66	ns.	1.37 ± 0.49

budesonide in the past	11/14 (78.5%)	p < 0.01	11/28 (39.28%)

systemic steroid ever	none		14/28 (50%)

actual immunosuppressants	none		9/28 (32.14%)

Subjects in the healthy control (HC) group had not been treated with medications containing steroids or immunosuppressants, and they were symptomless. The results are presented as mean ± standard deviation; Student's t-tests were applied for statistical analysis. (LC = lymphocytic colitis, CC = collagenous colitis, ns = not significant.)

### Bone density parameters

Low bone mass was detected in 57%, 46% and 10.7% of MC patients, CD patients and the HC group, respectively (Table 2.). Incidence of low bone mass was significantly lower in MC and CD patients than in the HC group (p < 0.01). One of 14 patients with MC had osteoporosis (t-score < -2.5), while seven had osteopenia (t-score < -1.0). Five CD patients had osteoporosis and 12 had osteopenia, according to the WHO criteria; three patients from the HC group had osteopenia. BMD was lower at the femoral neck in MC and CD patients than in healthy controls (HC).

**Table 2:** Major objective bone density parameters in microscopic colitis, Crohn's disease and healthy controls.

	MC	significance (MC vs. CD)	CD	HC
Femoral BMD (g/cm^2^)	0.852 ± 0.165**	ns.	0.807 ± 0.136**	1.056 ± 0.126

Lumbar BMD (g/cm^2^)	0.928 ± 0.156	p < 0.05	0.847 ± 0.112*	0.949 ± 0.112

Non-dominant radius BMD (g/cm^2^)	0.565 ± 0.093*	ns.	0.672 ± 0.056*	0.667 ± 0.072

Femoral t-score	-0.638 ± 1.437*	ns.	-0.607 ± 1.09	-0.211+1.053

Lumbar t-score	-1.203 ± 1.42	ns.	-1.390 ± 1.124	-1.328 ± 1.041

Radius t-score	-1.37 ± 1.135*	ns.	-1.090 ± 1.236*	-0.882 ± 1.106

(BMD: bone mineral density, MC: microscopic colitis, CD: Crohn's disease; HC: healthy controls; paired and independent sample Student's *t*-tests were applied. The results are presented as mean ± standard deviation; * = p < 0.05 and ** = p < 0.01 compared to HC, ns = not significant.)

There was no significant difference between the femoral neck BMD levels from MC and CD patients. Bone density of the lumbar spine in MC patients was lower than the HC group, but higher than CD patients. BMD measured at the non-dominant radius was lower in MC patients than in the HC group and CD patients. Femoral and radius t-score values were lower in MC patients than in controls (Table 3).

**Table 3:** Incidence of osteopenia and osteoporosis in microscopic colitis, Crohn's disease and healthy controls.

	MC	significance (MC vs. CD)	CD	HC
incidence of low bone mass	57%**	ns.	46%**	10,7%

osteoporosis	1 (7%)	ns.	5 (17.8%)	0

osteopenia	7 (50%)**	ns.	12 (42%)**	3 (10.7%)

(MC: microscopic colitis, CD: Crohn's disease; HC: healthy controls; paired and independent sample Student's *t*-tests were applied. The results are presented as mean ± standard deviation; * = p < 0.05 and ** = p < 0.01 compared to HC, ns = not significant.)

### Bone metabolism parameters

**Figure 1:**
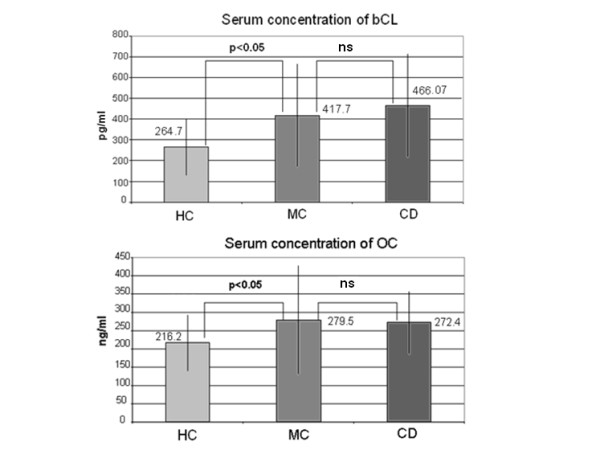
**Serum beta-crosslaps and osteocalcin concentrations were higher in microscopic colitis and Crohn's disease patients than in healthy controls**. (OC: osteocalcin, bCL: beta-crosslaps, MC: microscopic colitis, CD: Crohn's disease, HC: healthy control; paired and independent sample Student's *t*-tests were applied. The results are presented as mean ± standard deviation; ns: not significant.)

The direction of bone metabolism was evaluated by detecting the bone resorption and formation markers, bCL and OC (Figure 1). The mean bCL concentration was higher in MC patients and CD patients than in the HC group. The rate of bone resorption, reflected by the serum concentration of bCL, was more pronounced in CD patients. There was a negative correlation between the bCL concentration and the femoral and radius t-score values in MC patients (-0.8 and -0.77, respectively, p < 0.05) and CD patients (-0.83 and -0.79, respectively, p < 0.05). Significantly higher serum concentrations of the bone formation marker OC were measured in MC and CD patients than in the HC group. However, the mean concentration of OC was within the normal range in each group.

### Risk factors for low bone mass

**Figure 2:**
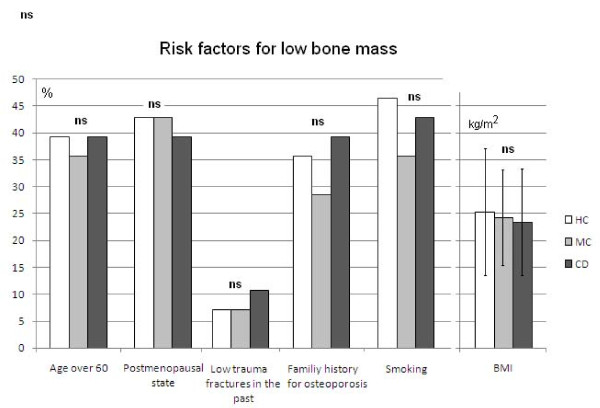
**Risk factors regarding low bone mass occur with a similar frequency among patients with Crohn's disease, microscopic colitis and healthy subjects**. Current steroid therapy is a risk factor for low bone mass, but this kind of medication was an exclusion criterion in this study. (MC: microscopic colitis, CD: Crohn's disease, HC: healthy control; paired and independent sample Student's *t*-tests were applied. The results are presented as percentage, BMD results are presented as mean ± standard deviation; ns: not significant.)

American College of Gastroenterology and American Gastroenterology Association guidelines recommend screening IBD patients with DEXA if they have one of the following risk factors: postmenopausal state, ongoing corticosteroid treatment, cumulative prior use of corticosteroids exceeding three months, history of low trauma fractures, or aged over 60 years [30,31]. CD and MC patients and HC subjects were monitored for these risk factors. Other risk factors including family history for low bone mass, smoking status and BMI were also evaluated. There was no significant difference in the BMI (23.45 ± 8.56 vs. 24.23 ± 7.89 and 25.34 ± 12.4 kg/m^2 ^in CD, MC and HC, respectively), smoking status (5/14, 12/28 and 13/28 in MC, CD and HC groups; respectively) and family history for osteoporosis (5/14, 11/28 and 13/28 in MC, CD and HC groups; respectively). Results of the risk assessment are presented in Figure 2.

**Figure 3:**
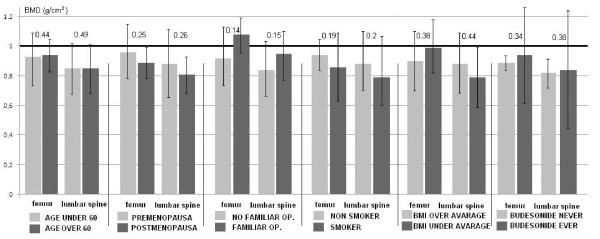
**Bone mass does not differ in microscopic colitis patients, with or without known risk factors for osteoporosis**. There was one low trauma fracture among patients with microscopic colitis; therefore, it was not possible to perform comparisons with regard to this risk factor. The black line signifies the normal value for bone mineral density. (BMD: bone mineral density, OP: osteoporosis; results are presented as g/cm^2^, numbers situated above the bars represent the level of significance between patients with or without risk factors. Paired and independent sample Student's *t*-test were applied for statistical analysis.)

There was no significant difference in the BMD of MC patients with or without associated risk factors (Figure 3). Previous steroid therapy is one of the most important risk factors for bone loss. Therefore, the bone densities of MC patients with or without previous short term budesonide therapy were compared. Lumbar and femoral BMD were similar in patients who had or had not been treated with budesonide (0.82 ± 0.09 g/cm^2 ^vs. 0.86 ± 0.43 g/cm^2^, ns; and 0.89 ± 0.04 vs. 0.99 ± 0.49 g/cm^2^, ns; respectively). Femoral and lumbar T- scores for budesonide naïve MC patients were low (-1.68 ± 0.16 and -1.7 ± 0.98).

The serum calcium concentrations of the MC, CD and HC groups (2.36 ± 0.08 mg/ml, 2.42 ± 0.11 mg/ml and 2.39 ± 0.09 mg/ml) remained unchanged, and the parathyroid hormone level was in the normal range in patients with MC (36.64 pg/ml).

## Discussion

There are no available data regarding alterations in bone metabolism as a complication of MC. The results presented herein suggest decreased bone density in a cohort of MC patients with no clinical signs of malabsorption.

Bone mass was decreased to the same extent in the MC group and the CD group that had been matched for age, gender and menopausal status. Considering the similarly decreased BMD ratios in the MC and CD groups (57% vs. 46% respectively), and taking into consideration that the incidence of MC is underestimated in daily clinical practice, it is proposed that low bone mass is a common occurrence in patients with undiagnosed MC who suffer from chronic diarrhoea.

Bone density parameters of patients with MC were independent from coexisting risk factors including previous short term budesonide therapy. Based on these data, a common pathogenic factor is proposed for MC and the associated bone loss.

The pathophysiology of low bone mass in gastrointestinal diseases is different from senile or postmenopausal osteoporosis. Accelerated bone resorption - so-called uncoupling - is responsible for low bone mass in CD [32]. It has been demonstrated that serum markers for bone formation and bone resorption are higher in CD than HC [33]. The ratio of bone resorption and bone formation markers was similar in MC and CD patients. Serum concentrations of bCL were doubled in MC and CD patients compared with the HC group. Compensatory elevation of the bone formation marker OC was more modest in MC and CD patients than the HC group, without exceeding the normal range. There was a negative correlation between the bCL concentration and femoral or radius t-score values in MC patients, demonstrating a pathogenic relationship between DEXA and the laboratory measurements.

Bone mineral content was significantly lower in parts of the skeleton with a high cortical versus trabecular bone ratio in MC patients; BMD was more profoundly decreased in the femoral neck and non-dominant radius than in the lumbar spine. The mineral content of these regions was similar in MC and CD patients in the present study; however, a lower BMD was evident at the lumbar spine of CD patients.

Thyroid and parathyroid hormones can affect the two compartments of bone differently: the loss of cortical bone is characteristic of hyperthyroidism and hyperparathyroidism. Normal levels of thyroid stimulating hormone, free thyroid hormones, serum calcium and parathyroid hormones exclude a role for these factors in cortical bone loss of the patients in this study.

No data are available to describe the various regulation of bone homeostasis in the cortical and trabecular bones of IBD patients. However, it is known that factors including sex hormones [34] and their receptors [35] have different impacts on cortical and trabecular bones. Hypogonadism is a complication of inflammatory bowel disease, but a pathogenic role for gonad dysfunction has not been proven in regard to the altered bone metabolism in IBD [36,37].

A major limitation of this study is the low number of MC patients; there was no possibility of performing subgroup analysis in this small cohort. Data concerning LC and CC patients were analyzed together as histology is the only relevant difference between these two types of MC. Furthermore, the mean age of patients with MC is similar to the perimenopausal age and to the first postmenopausal decade; therefore, low BMD in patients with chronic diarrhoea is not a specific indicator of definitive colonic disease. It is suggested that patients with chronic diarrhoea undergo a biopsy during colonoscopy; a specific request to the pathologist should be made to consider the diagnosis of MC. Densitometry should only be performed on patients with histologically confirmed MC.

## Conclusion

The present study demonstrates that bone loss can be an important problem in MC. A similarly decreased BMD was observed in patients with MC and CD. Low bone mass was detected in the femur and radius, and these bones contain more cortical than trabecular bone. Uncoupled bone remodelling was demonstrated in MC, with bone resorption demonstrated to exceed compensatory bone formation. The current findings are similar to the changes observed in bone homeostasis in CD.

## Competing interests

The authors declare that they have no competing interests.

## Authors' contributions

All authors collected data regarding their microscopic colitis patients and participated in data processing. GL, KL and PM actively participated in the design of the trial and database construction. Statistical analysis was performed by PM and PLL, KL, ZT and PM prepared the manuscript. All authors read and approved the final manuscript.

## Pre-publication history

The pre-publication history for this paper can be accessed here:

http://www.biomedcentral.com/1471-230X/11/58/prepub
